# Adaptive Discriminative Regions Learning Network for Remote Sensing Scene Classification

**DOI:** 10.3390/s23020773

**Published:** 2023-01-10

**Authors:** Chuan Tang, Xiao Zheng, Chang Tang

**Affiliations:** 1 School of Computer Science, China University of Geosciences, No. 68 Jincheng Road, Wuhan 430078, China; 2 School of Computer, National University of Defense Technology, Deya Road, Changsha 410073, China

**Keywords:** remote sensing, scene classification, deep convolutional neural networks, RSSC, DCNNs

## Abstract

As an auxiliary means of remote sensing (RS) intelligent interpretation, remote sensing scene classification (RSSC) attracts considerable attention and its performance has been improved significantly by the popular deep convolutional neural networks (DCNNs). However, there are still several challenges that hinder the practical applications of RSSC, such as complex composition of land cover, scale-variation of objects, and redundant and noisy areas for scene classification. In order to mitigate the impact of these issues, we propose an adaptive discriminative regions learning network for RSSC, referred as ADRL-Net briefly, which locates discriminative regions effectively for boosting the performance of RSSC by utilizing a novel self-supervision mechanism. Our proposed ADRL-Net consists of three main modules, including a discriminative region generator, a region discriminator, and a region scorer. Specifically, the discriminative region generator first generates some candidate regions which could be informative for RSSC. Then, the region discriminator evaluates the regions generated by region generator and provides feedback for the generator to update the informative regions. Finally, the region scorer makes prediction scores for the whole image by using the discriminative regions. In such a manner, the three modules of ADRL-Net can cooperate with each other and focus on the most informative regions of an image and reduce the interference of redundant regions for final classification, which is robust to the complex scene composition, object scales, and irrelevant information. In order to validate the efficacy of the proposed network, we conduct experiments on four widely used benchmark datasets, and the experimental results demonstrate that ADRL-Net consistently outperforms other state-of-the-art RSSC methods.

## 1. Introduction

Remote sensing scene classification (RSSC) aims to classify a remote sensing (RS) scene into a certain category based on the content of a given remote sensing image, which has widely potential applications such as land management [1,2], urban planning [3], wild fires, crop growth monitoring [4,5,6], and target detection [7]. Due to the high variance of the distance between earth and the imaging sensors, RS images are often also with large-scale variance, which results in various challenges to RSSC.

During the past decades, a large number of methods have been proposed for the task of RSSC. Based on the feature representation manner, these methods can be classified into two major categories, i.e., traditional hand-crafted feature based methods and deep learning based methods [8]. For the first category, commonly used features such as scale-invariant feature transform [9], GIST [10], histogram of oriented gradients [11], and local binary patterns (LBP) [12,13] are first extracted from RS images, then a classifier is trained on a kind of extracted feature or multiple features [14,15,16]. Although the hand-crafted features are with good physical interpretation, they are often time-consuming and need professional domain knowledge to produce. In addition, the representation ability of traditional features is also limited [17].

**Figure 1:**
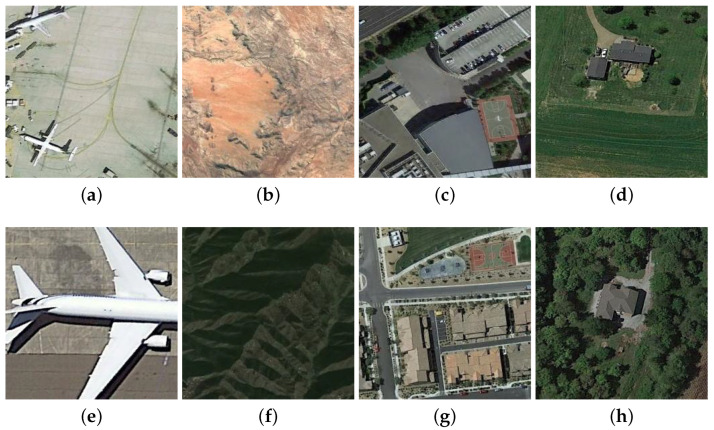
Some challenging cases in RSSC. For each column of image pair, they belong to the same class of scene, but (**a**,**e**) have high scale variability between objects, (**b**,**f**) have absolutely different appearance, (**c**,**g**) have a large number of noisy areas that interfere the discriminative regions, and (**d**,**h**) large redundant areas.

**Figure 2:**
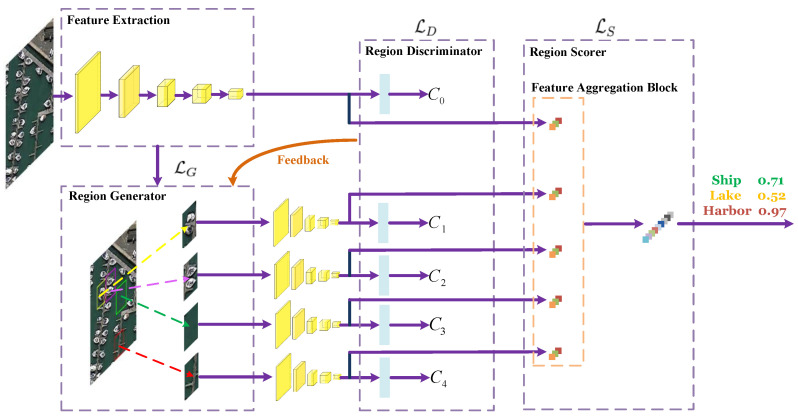
Flowchart of our ADRL-Net designed for remote sensing image classification (N=4).

Due to the powerful feature representation and learning capability, deep convolutional neural networks (DCNNs) [18,19] have made a breakthrough for many computer vision tasks, such as image classification [20], object detection [21,22], image restoration [23,24], and semantic segmentation [25,26]. In the past few years, there are also many DCNNs-based methods proposed for RSSC [27,28,29,30,31,32,32]. As straightforward approaches, many existing CNNs such as AlexNet [33], VGGNet [20], and GoogLeNet [34] are directly transferred to the RS scene classification task [29,35,36,37,38]. In this kind of methods, only the feature maps of the last network layer are used for classification, while the feature maps of the lower hierarchical layers are not fully exploited. Therefore, in order to make full use of the multiscale deep features extracted from different layers, many feature aggregation methods have been put forward, which first extract hierarchical deep features by using a certain pretrained CNN structure. Then, the extracted feature maps are encoded by designed feature aggregation branch to capture the high-level semantic information and low-level appearance details of a given image. Although great success of RSSC has been achieved by feature aggregation networks, they treat the RS scene as a whole. Therefore, the classification performance could be significantly affected by some redundant and noisy regions of the RS image. In addition, the objects that dominantly determine the category of a RS image often have high size variance, e.g., the aircraft in an airport are usually with different sizes due to different models or different distances between the imaging sensors and earth land. In Figure 1, we give some examples about the challenging cases in RSSC. In order to solve these issues, we propose a deep neural network (ADRL-Net) that adaptively learns discriminative regions to promote RSSC. Our proposed ADRL-Net consists of a region generator, a region discriminator, and a region scorer, and it selects the most informative image regions in a self-supervised manner. Meanwhile, noisy and redundant regions are effectively excluded. Figure 2 gives a brief structure of the proposed network.

In summary, the technical contributions of this work are as follows:

- We propose an adaptive discriminative regions learning network (ADRL-Net) for remote sensing scene classification, which can effectively generate informative regions as well as reduce unimportant/redundant regions from a RS image for classification. The proposed ADRL-Net is composed of three main modules, including a region generator, a region discriminator, and a region scorer.
- We design a self-supervision mechanism to adaptively locate discriminative regions by using the region generator and the region discriminator. During the training process, the three main modules of ADRL-Net can cooperate with each other to learn the optimal parameters for testing.
- Experiments with extensive effectiveness analysis on four benchmark datasets are conducted to validate the efficacy of the proposed network.

## 2. Related Work

As discussed in previous sections, RSSC works can be generally classified into traditional hand-crafted feature-based methods and deep learning based methods. Since our proposed method is based on DCNNs, we just only introduce some related DCNNs-based RSSC methods in this section. For traditional hand-crafted feature based methods, one can refer to [39,40,41,42,43,44,45].

Over the past decade, DCNNs has achieved great success in computer vision community [19,20,46]. As a special subfield of computer vision, remote sensing scene classification has also been pushed forward with a big step by DCNNs and many successfully works have been proposed. In [30,32,47,48,49], Zhang et al. extracted a representative set of patches from the salient regions in original image data set, then the patch set is feed into a sparse autoencoder to learn a set of feature extractors for scene classification. Based on pretrained network models on ImageNet [50], many DCNNs-based networks are designed for RSSC by fine-tuning on remote sensing image datasets. By embedding different CNN model-based feature extractors and integrating them with various feature encoding methods, Hu et al. [35] transferred DCNNs for the scene classification of high-resolution remote sensing images. The bag of visual words model is also aggregated with the convolutional activation layer for RSSC [41]. Instead of using traditional hand-crafted features, the DCNNs is directly used to extract high-level features for remote sensing image representation [51,52], and deep features are also combined with hand-crafted features to boost the classification performance [53]. By designing a covariance pooling method, He et al. [54] combined the different layers of pretrained CNN models to enhance the representation ability of deep features for classifying challenging remote sensing images. For solving the large-scale variance problem in remote sensing scene images, the skip connections are added to combine the multiresolution feature maps together [55]. In [36], Liu et al. designed a Siamese CNN to combine the identification and verification models of CNNs with a metric learning regularization term for tackling some challenging cases in RSSC, such as lack of rich label information, small scale of scene classes, and lack of image diversity. Since little attention has been paid for exploring the semantic label information for feature aggregation, Lu et al. [56] proposed a supervised convolutional features’ encoding module and a progressive aggregation strategy to aggregate the intermediate features for effective scene representation of remote sensing scene images. By considering that the multilayer convolutional features are usually treated equally with the hierarchical structure of features being ignored, a gated bidirectional network is developed to integrate the hierarchical feature aggregation and the interference information elimination into an end-to-end network [57]. In order to enhance the effects of representative objects and feature channels, spatial self-attention and channel-attention-based deep feature fusion mechanisms are also utilized [58,59,60,61]. Wang et al. [59] designed a granular framework which allows progressively cropping the input image to learn multigrained features and automatically captures the latent ontological structure of remote sensing datasets.

## 3. Methodology

In this section, we first give a brief introduction of the proposed network, i.e., ADRL-Net. Then, the details of each network component will be elaborated.

### 3.1. Overview of the Proposed ADRL-Net

Generally, the motivation behind our network is that informative regions are more important to represent an remote sensing image scene; thus, these regions should be more discriminative for RSSC. For example, if we can separate the basketball courts from the images in the third column of Figure 1, then the affects from the surrounding buildings can be effectively suppressed and the whole images can be easily classified correctly. However, it is laborious to mark the discriminative regions for each image, how to adaptively learn those regions is a critical problem. To this end, we design an adaptive discriminative regions learning network to pick out those informative regions from a remote sensing image for boosting the performance of RSSC. There are three main modules in our proposed ADRL-Net, including a discriminative region generator which produces a bunch of alternative region proposals, a region discriminator which filters out some informative regions from the proposals according to their class-aware confidence, and a region scorer which classifies the remote sensing scene based on the features extracted from original image and those discriminative regions. During the learning process, the confidences of proposals obtained from the region discriminator module are backpropagated to the region generator module for helping learning more accurate region proposals. When the network gradually converges, the top-*N* informative regions produced by region generator are input to the region scorer module for final scene classification. Since both the features extracted from the whole image and discriminative regions are important for final classification, we design a feature aggregation block to fuse the features from the whole image and the top-*N* regions. In such a manner, both the global context information and local region-aware specificity of an image can be well captured. Following, we give the details of each module and component of the ADRL-Net.

### 3.2. Discriminative Region Generator

The task of discriminative region generator is to generate some regions proposals that could be informative and important for classification, which is similar to previous region proposal problem [62,63,64]. In order to get region proposals as well as their corresponding informativeness, we borrow the idea of the anchor-based region proposal network which shares convolutional layers with the classifier and generate proposals by mitigating the marginal cost [65]. Inspired by the idea of anchors, we input an remote sensing scene image into the discriminative region generator and producing *M* alternative rectangle regions $\left\{r_{1},r_{2},\cdots ,r_{M}\right\}$, and each region is assigned a score which denotes its informativeness for final classification. For a given image with size W×H, the scales and ratios of its anchors are set to {min(W,H)min(W,H)1212,min(W,H){min(W,H)min(W,H)1212,min(W,H)66,min(W,H)min(W,H)33} and {1:1,3:2,2:3}, respectively. Supposing the informativeness of all anchors are denoted as $\left\{I\left(r_{1}\right),I\left(r_{2}\right),\cdots ,I\left(r_{M}\right)\right\}$, then we can sort the information list in a decent or ascend order.

### 3.3. Region Discriminator

Since the region proposals produced by the discriminative region generator are usually noisy and redundant, we need to choose the most informative ones. By adopting the nonmaximum suppression (NMS) [66] on the proposals based on their informativeness, we can roughly pick up top-*K* proposals and feed them into the region discriminator to obtain their confidence as $\left\{C\left(r_{1}^{{}^{\prime }}\right),C\left(r_{2}^{{}^{\prime }}\right),\cdots ,C\left(r_{K}^{{}^{\prime }}\right)\right\}$. In our network, we optimize discriminative region generator module to constrain that $\left\{I\left(r_{1}^{{}^{\prime }}\right),I\left(r_{2}^{{}^{\prime }}\right),\cdots ,I\left(r_{K}^{{}^{\prime }}\right)\right\}$ and $\left\{C\left(r_{1}^{{}^{\prime }}\right),C\left(r_{2}^{{}^{\prime }}\right),\cdots ,C\left(r_{K}^{{}^{\prime }}\right)\right\}$ have the same order by using pairwise ranking loss function [67,68]. Specifically, supposing that $\Psi (I\left(r_{i}^{{}^{\prime }}\right),I\left(r_{j}^{{}^{\prime }}\right))$ is a nonincreasing function that encourages $I\left(r_{i}^{{}^{\prime }}\right)>I\left(r_{j}^{{}^{\prime }}\right)$ if $C\left(r_{i}^{{}^{\prime }}\right)>C\left(r_{j}^{{}^{\prime }}\right)$. Then, the loss on the region informativeness and confidence of all sample pairs are defined as follows:

(1) $$\begin{array}{cc} \; & L\left(\Psi ,I\left(r_{k}\right),C\left(r_{k}\right)\right)=\sum_{\left(i,j\right):C\left(r_{i}\right)<C\left(r_{j}\right)}\Psi (I\left(r_{i}\right),I\left(r_{j}\right))\\ \; & k=1,\cdots ,K. \end{array}$$

As to the region discriminator module, it is optimized by minimizing the cross-entropy loss between ground-truth class and the predicted confidence.

In this work, we adopt NMS to choose the informative regions from region proposals. In detail, we leverage the region proposal network used in Faster RCNN [65] to get the informativeness I(r) of local regions. As to the region confidence C(r), we input the extracted feature of each region proposal to a fully connected layer for approximating the mapping C:A→[0,1] which denotes the confidence of each region, as shown in Figure 2.

### 3.4. Region Scorer

**Figure 3:**
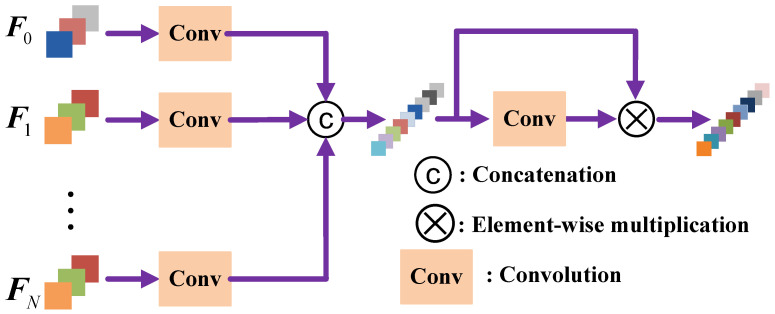
The detailed structure of the designed feature aggregation block.

When the discriminative region generator module and region discriminator gradually converges, we can obtain some important regions that help the classifier discriminate remote sensing scene images from different classes. In our network, we choose the top-*N* regions produced by the discriminative region generator and adjust them to a predefined size; then, the resized rectangular regions are fed into feature extractor to generate their corresponding feature vectors. Finally, the features extracted from original image and the top-*N* regions are combined together and feed into a fully connected layer for classification. It should be noted that different discriminative regions could contribute different to the final classification performance, we design and embed a feature aggregation block in region scorer before classification to fuse the features from the whole image and the top-*N* regions with different weights. In Figure 3, we show the detailed structure of the proposed feature aggregation block. The feature extracted from original image and the *N* discriminative regions are denoted as $F_{0}$ and $F_{1},\cdots ,F_{N}$, respectively. Since $F_{0}$ captures more global context information while $F_{1},\cdots ,F_{N}$ focus on different local objects/areas, we add a convolution operation in each feature branch for feature adaptation before concatenating them together. Then, for each feature branch, the adapted features can be calculated as:

(2) $$F_{i}^{{}^{\prime }}=W_{i}*F_{i}+b_{i},$$

where * represents convolution operation; $W_{i}$ and $b_{i}$ are the weights and bias of the convolution that need to be learned during training. Then, the final fused features can be calculated as:

(3) $$F=CatF⊗(W^{\prime }*CatF+b^{\prime }),$$

where ⊗ denotes the Hadamard product (elementwise multiplication), $W^{\prime }$ and $b^{\prime }$ are the weights and bias of the feature aggregation learning process, CatF denotes the concatenated adapted features of different discriminative regions, which is obtained by $CatF=Cat(F_{0}^{{}^{\prime }},F_{1}^{{}^{\prime }},\cdots ,F_{N}^{{}^{\prime }})$, where Cat represents the concatenation operation.

### 3.5. Loss Function and Network Optimization

Since there are three main modules in our network, the loss function of the whole network also consists of three term, i.e., discriminative region generation loss $L_{G}$, region discriminator loss $L_{D}$ and region scorer loss $L_{S}$.

#### 3.5.1. Discriminative Region Generation Loss

As mentioned in previous section, we use the pairwise ranking loss to optimize the discriminative region generator module. Then, the form of the general loss for this module is defined as Equation (1). In this work, we use the hinge loss to define the nonincreasing function in our experiments. Therefore,

(4) $$L_{G}=\sum_{\left(i,j\right):C\left(r_{i}\right)<C\left(r_{j}\right)}\max(1-\left(I\left(r_{i}\right)-I\left(r_{j}\right)\right),0).$$

As can be seen, the loss function $L_{G}$ encourages that $I(r_{i})$ and $C(r_{i})$ are in the same order.

#### 3.5.2. Region Discriminator Loss

The region discriminator loss is defined as the commonly used cross-entropy function, which is formulated as follows:

(5) $$L_{D}=-\sum_{i=0}^{N}\log M(r_{i}),$$

where M denotes the confidence function that maps the discriminative regions ($r_{1},\cdots ,r_{N}$) and original image ($r_{0}$) to its probability being ground-truth class.

#### 3.5.3. Region Scorer Loss

The main task of region scorer is classifying a certain remote sensing scene image based on the features extracted from those discriminative regions and original images. Therefore, we also use the cross-entropy as the loss function for this module, and we define

(6) $$L_{S}=-\log F\left(r_{0},r_{1},\cdots ,r_{N}\right),$$

where F makes the final classification results based on fused features.

Finally, the total loss of the whole network is defined as:

(7) $$L=L_{G}+\alpha L_{D}+\beta L_{S}.$$

where α and β are hyperparameters to balance different loss terms. In our experiments, we empirically set all the hyperparameters in Equation (7) to 1 and set N=6.

It should be noted that the working mechanism our proposed ADRL-Net is similart to the idea of generative adversarial networks (GAN) [69,70]. However, there are several different points of our proposed ADRL-Net as follows:

- Different to original GAN that generate new data, we just choose some discriminative regions from the region proposals that are produced in advance.
- The top-*K* discriminative regions are picked up by adopting the nonmaximum suppression (NMS) on the region proposals based on their informativeness, rather than generating new data.
- The loss functions are different to original GAN. As shown in our original paper, the loss function of contains three parts, i.e., discriminative region generation loss ($L_{G}$), region discriminator loss ($L_{D}$), and region scorer loss ($L_{S}$). Therefore, the loss function of our proposed ADRL-Net is totally different to original GAN, which means that the learning mechanism of our proposed ADRL-Net is different to original GAN.

## 4. Experiments

In this section, we give the experimental results of our ADRL-Net. In order to demonstrate its efficacy for RSSC, we also compare it with several other state-of-the-art methods.

### 4.1. Datasets

We use four popular remote sensing scene image datasets in our experiments. The details are as follows:

- Aerial Image Dataset (**AID**) [71] contains 10,000 images which are from 30 classes. The number of images of each class ranges from 220 to 420, with a size of 600×600 pixels in RGB space. The spatial resolution of the images varies from about 8 m to 0.5 m.
- UC Merced Land Use (**UC Merced**) dataset [40] consists of 2100 images captured from 21 scene categories. There are 100 images with a size of 256×256 pixels in RGB color space for each class and the pixel resolution of each image is one foot.
- NWPU-RESISC45 (**NWPU**) dataset [41] contains 31,500 images with 45 scene classes. It is one of the largest datasets available for evaluation of RSSC. There are 700 images with a size of 256×256 pixels in RGB color space for each class. For most of the images, the spatial resolution varies from about 30 to 0.2 m/pixel. There are a series of challenging cases for RSSC task in this dataset, such as large-scale image variations, highly interclass similarity, and within-class diversity.
- **WHU-RS19** dataset [72] consists of 19 scene classes and each class contains 50 images at least. The images are extracted from Google Earth imagery directly with image size 600×600.

For the training images of each dataset, we adopt random horizontal flipping with 50% probability for data augmentation.

### 4.2. Implementation Details and Experimental Settings

In our experiments, we use three popular CNN structures, i.e., AlexNet, VGG16 and ResNet50, as the feature extraction backbone to construct our ADRL-Net, respectively. For each CNN structure backbone, the features before the last fully connected (FC) layer are used as input to the region discriminator and region scorer modules. We use Momentum Stochastic Gradient Descent (SGD) algorithm to optimize the network, and the initial learning rate is set to 0.001 and multiplied by 0.1 after 10 epochs, the weight decay is set to 1 $\times 10^{-4}$. The NMS threshold used for picking up top-*K* proposals as input to the region discriminator is set to 0.3. Based on the empirically experimental results, the whole network is not sensitive to the hyperparameters. Therefore, the experimental results reported in following sections are with the parameter values mentioned above. Since we will use random sampling to generate training and testing image sets, five times of training and testing are carried out for each dataset, and the average and standard deviation (Std) of the overall accuracy (OA) after five runs are reported. The Pytorch implementation of the network will be publicly released.

### 4.3. Experimental Comparison with Other Methods

To verify the superiority of our proposed ADRL-Net, we compare its performance with the following ten methods:

- Fine-tuned AlexNet and VGG16. We replace the last FC layer of the CNN structures with a randomly initialized layer with specified output dimension that equals to the number of categories;
- VGG-M [51], which uses the VGG net as the feature extractor, and two FC layers are embedded to obtain the final features used for classification with linear SVM.
- BoVW [41], which generates visual words from deep convolutional features using off-the-shelf convolutional neural networks;
- DFF [51], which is a deep feature fusion network for RSSC;
- MSCP [54], it can naturally combine multilayer features which are obtained through a pretrained CNN model.
- MCNN [73], it is a multiscale CNN model and constructs a fixed-size and a variable-size CNN, which could solve the issue of large scale variation in RSSC.
- DCNN [74], it is a discriminative CNN model, the metric learning in which is combined with a CNN model to enhance the discrimination of images from different classes.
- ARCNet [75], which is an end-to-end attention recurrent convolutional network for scene classification with the guidance of the human visual system.
- SCCov [55], skip-connected covariance network, which is a improved version of MSCP and embeds kip connections and covariance pooling into one network.
- GBNet [57], gated bidirectional network, which integrates the hierarchical feature aggregation and the interference information elimination into an end-to-end network for RSSC.

#### 4.3.1. Experiments on AID Dataset

**Figure 4:**
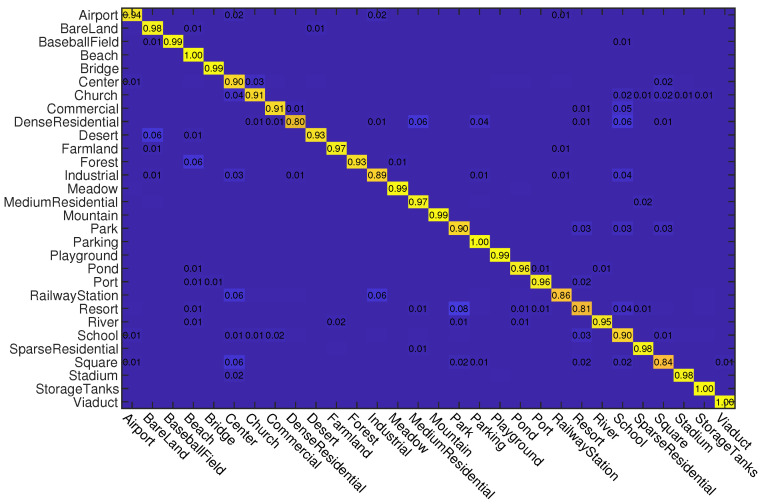
Confusion matrices obtained by ADRL-Net with ResNet50 as backbone on AID dataset. Tr = 20%.

**Figure 5:**
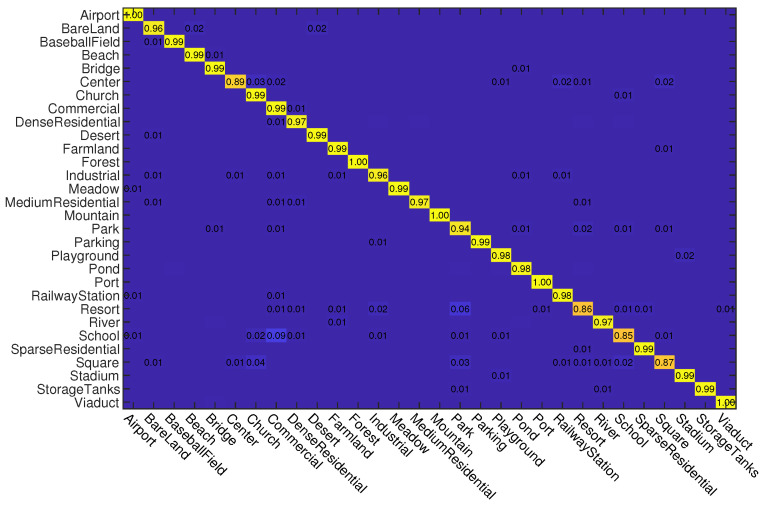
Confusion matrices obtained by ADRL-Net with ResNet50 as backbone on AID dataset. Tr = 50%.

First, we conduct experiments on the AID dataset. Similar to the settings in previous works [55,71], we use two kinds of data splits for training and testing. For the first split, 20% samples of each class are randomly selected for training and the rest are used for testing. For the second split, 50% samples of each class are randomly selected for training and the rest are used for testing. In Table 1, we give the OA of different methods on this dataset. As can be seen, when the training rate (Tr) is 20%, the proposed ADRL-Net outperforms all of other compared methods by using both ResNet50 and VGG16 as backbone structures. It can reach 94.24% and 93.67% OA when the backbone network structures are ResNet50 and VGG16, respectively. When Tr = 50%, although ADRL-Net does not perform the best when it uses VGG16 as the backbone network, its performance is still competitive excluding DCNN. In addition, we also show the confusion matrices obtained by ADRL-Net with ResNet50 as backbone in Figure 4 and Figure 5. As can be seen, some classes such as center, resort, school, and square are difficult to recognize, which is also a common problem for other methods. This is because that there are many different and noisy objects in these categories. It should be noted that our ADRL-Net can reach 100% classification accuracy for those categories which are composed by specific objects, such as airport, beach, forest, mountain, port, and viaduct.

#### 4.3.2. Experiments on UC Merced Dataset

**Figure 6:**
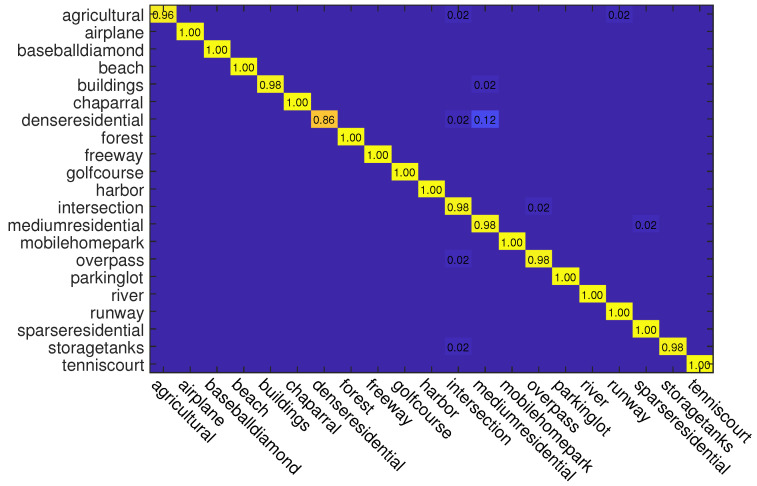
Confusion matrices obtained by ADRL-Net with ResNet50 as backbone on UC Merced dataset. Tr = 50%.

**Figure 7:**
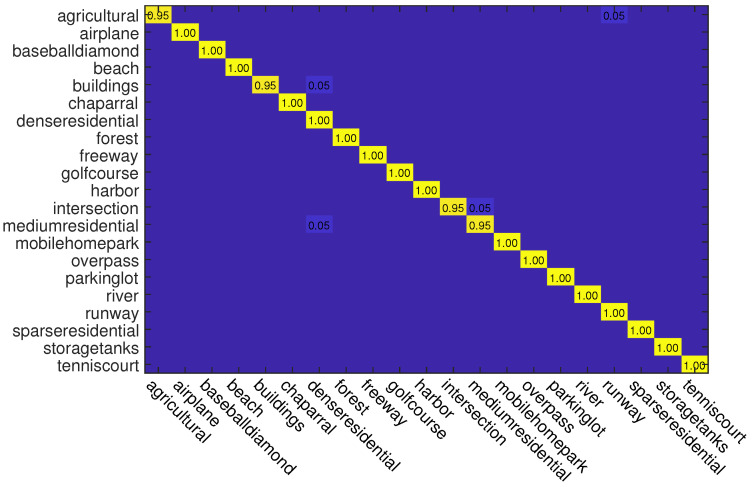
Confusion matrices obtained by ADRL-Net with ResNet50 as backbone on UC Merced dataset. Tr = 80%.

In this experiment, we also use two kinds of data splits for training and testing. For different splits, 50% and 80% samples of each class are randomly selected for training and the rest are used for testing, respectively. The classification accuracies of different methods on this dataset are reported in Table 2. As can be seen, when the training rate is 50%, ADRL-Net with ResNet50 structure outperforms other methods with OA of 98.72%. When the backbone is VGG16, ADRL-Net also reaches 97.31% OA. In addition, we also show the confusion matrices obtained by ADRL-Net with ResNet50 as backbone in Figure 6 and Figure 7.

#### 4.3.3. Experiments on NWPU Dataset

**Figure 8:**
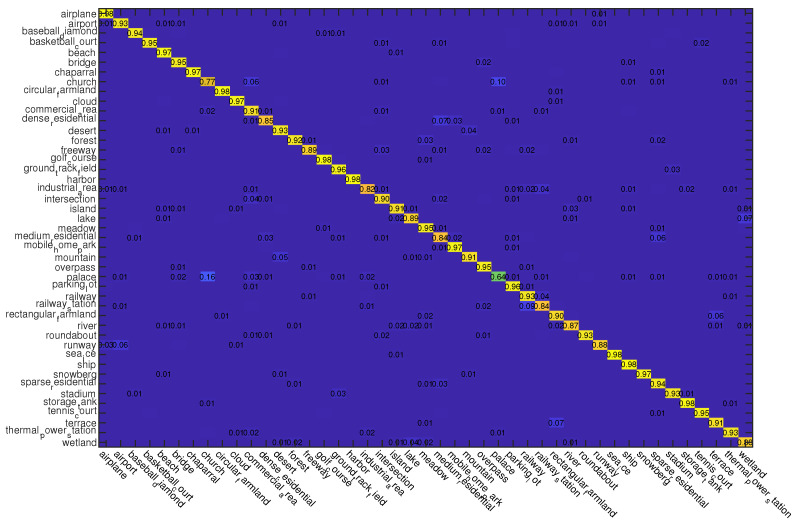
Confusion matrices obtained by ADRL-Net with ResNet50 as backbone on NWPU dataset (Picture can be enlarged by scaling the document). Tr = 10%.

**Figure 9:**
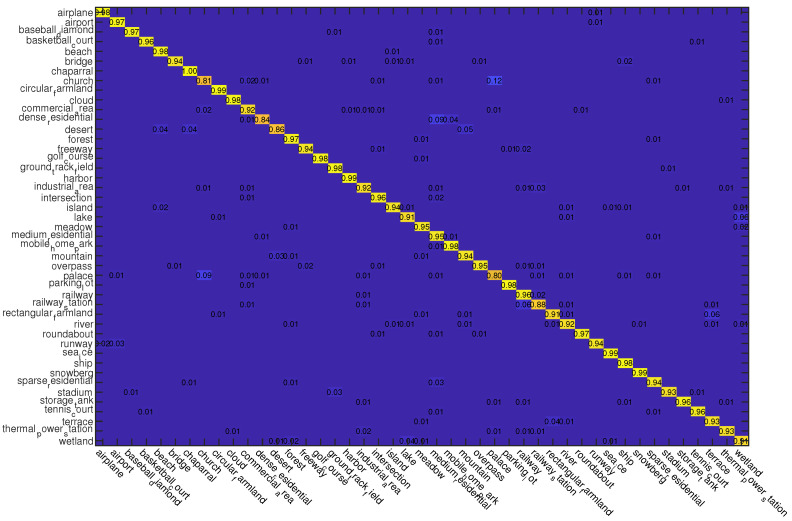
Confusion matrices obtained by ADRL-Net with ResNet50 as backbone on NWPU dataset (Picture can be enlarged by scaling the document). Tr = 20%.

For this dataset, we also use two kinds of data splits for training and testing, i.e., 10% and 20% samples of each class are randomly selected for training and the rest are used for testing, respectively. The classification results of different compared methods on this dataset are shown in Table 3. From the results, we can observe that the proposed ADRL-Net performs the best no matter what the training rate is. The confusion matrices obtained by ADRL-Net with ResNet50 as backbone are shown in Figure 8 and Figure 9.

#### 4.3.4. Experiments on WHU-RS19 Dataset

**Figure 10:**
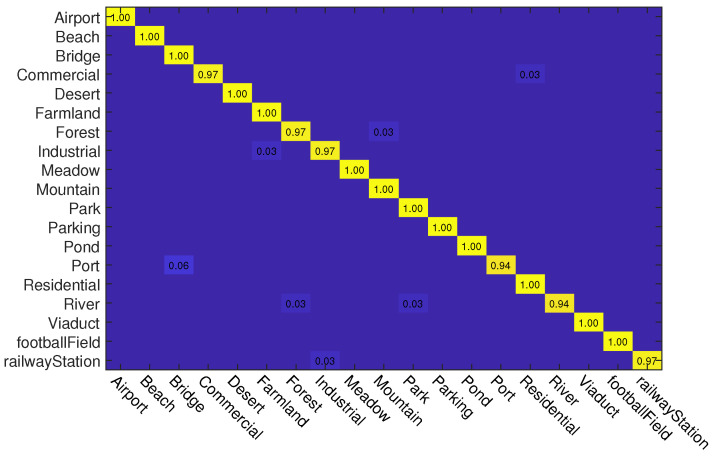
Confusion matrices obtained by ADRL-Net with ResNet50 as backbone on the WHU-RS19 dataset. Tr = 40%.

**Figure 11:**
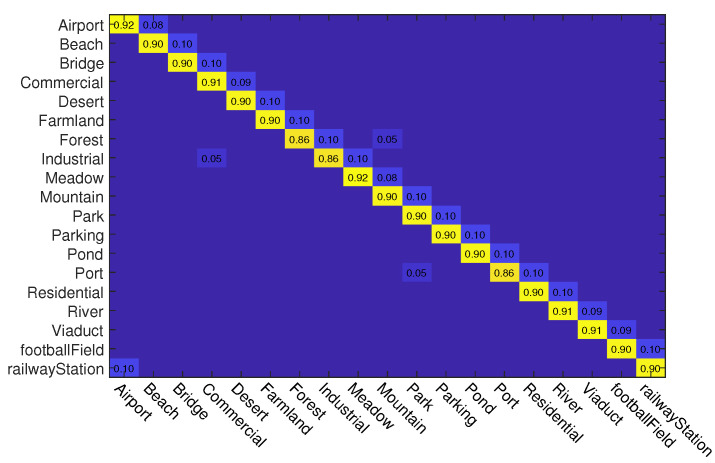
Confusion matrices obtained by ADRL-Net with ResNet50 as backbone on the WHU-RS19 dataset. Tr = 60%.

As to this dataset, 40% and 60% samples of each class are randomly selected for training and the rest are used for testing, respectively. We report the classification results of different methods on this dataset in Table 4. As can be seen from the table, although nearly all of the methods can reach more than 95% OAs, our proposed ADRL-Net still makes effective improvement. The confusion matrices obtained by ADRL-Net with ResNet50 as backbone are shown in Figure 10 and Figure 11.

### 4.4. Effectiveness Analysis of the Proposed ADRL-Net

**Figure 12:**
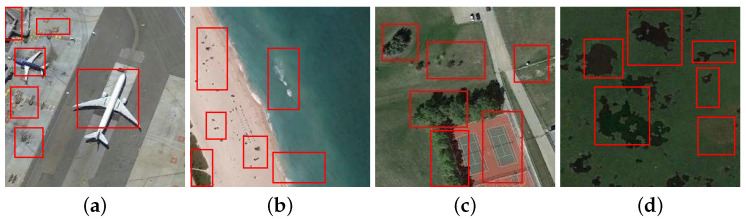
Some visual examples that show the discriminative regions obtained by ADRL-Net. (**a**) Airport; (**b**) Beach; (**c**) Tennis court; (**d**) Wetland.

In order to give an intuitive efficacy validation of the proposed network, we display some visual examples in Figure 12 that show the discriminative regions generated by the region generator module of ADRL-Net. As can be seen, the discriminative regions related to specific category can be effectively detected, e.g., in Figure 12b, the sand and seawater areas, which constitute the class of beach, are correctly highlighted.

### 4.5. Network Convergence Property

**Figure 13:**
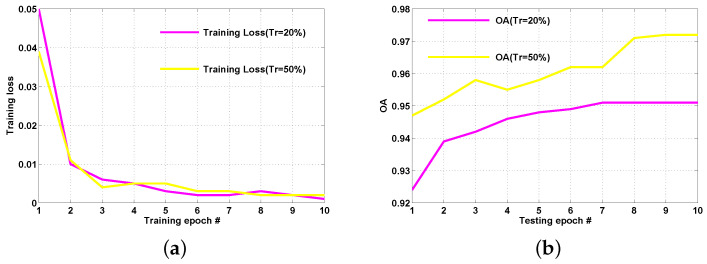
(**a**) Training loss of each epoch on AID dataset. (**b**) Corresponding OA of each epoch on AID dataset.

In our experiments, we end the training process of our network on each dataset after 10 epochs. In order to demonstrate the convergence property of ADRL-Net, we plot the training loss and the corresponding OA of each epoch on the AID dataset in Figure 13a,b, respectively. As can be seen, the whole network converges well in 10 time of training epochs and the corresponding OA goes steadily after 8 times of epochs.

## 5. Conclusions

In this work, we introduce a network named ADRL-Net for RSSC by adaptively learning discriminative regions from a given remote sensing image. There are three main modules in ADRL-Net including discriminative region generator, region discriminator, and region scorer. The three parts cooperate with each other to extract informative regions from the input image. Since the local informative regions focus on the specific objects or areas, we combine the features of the whole image and those discriminative regions to capture both global context information and local region information for final classification. Experiments on four widely used datasets are conducted to demonstrate the efficacy of the proposed ADRL-Net.

Since our proposed ADRL-Net aims to choose some discriminative regions from region proposals produced in advance for boosting the features used for final classification. Therefore, when a scene contains no informative regions (i.e., all the regions of the scene are background), the chosen regions will make no significant sense to final classification performance. In this case, we just need the features extracted from the whole scene for classification. In the future work, we aim to design a mechanism which can judge whether there are informative regions in a certain scene during the learning process.

## Figures and Tables

**Table 1:** OA comparison of different methods on AID dataset. The best two results are highlighted in bold font.

Backbone	Method	OA (Tr = 20%)	OA (Tr = 50%)
AlexNet	Fine-tuning	85.56 ± 0.32%	92.02 ± 0.22%
VGG16	Fine-tuning	90.53 ± 0.16%	95.03 ± 0.26%
VGG16	VGG-M	–	91.86 ± 0.28%
AlexNet	MSCP	88.99 ± 0.38%	92.36 ± 0.21%
VGG16	MSCP	91.52 ± 0.21%	94.42 ± 0.17%
AlexNet	MCNN	–	91.80 ± 0.22%
AlexNet	DCNN	85.62 ± 0.10%	94.47 ± 0.12%
VGG16	DCNN	90.82 ± 0.16%	**96.89 ± 0.10%**
AlexNet	SCCOV	91.10 ± 0.15%	93.30 ± 0.13%
VGG16	SCCOV	93.12 ± 0.25%	96.10 ± 0.16%
VGG16	GBNet	92.20 ± 0.23%	95.48 ± 0.12%
AlexNet	ADRL-Net	91.76 ± 0.18%	93.64 ± 0.15%
VGG16	ADRL-Net	**93.67 ± 0.18%**	95.83 ± 0.16%
ResNet50	ADRL-Net	**94.24 ± 0.17%**	**97.13 ± 0.14%**

**Table 2:** OA comparison of different methods on UC Merced dataset. The best two results are highlighted in bold font.

Backbone	Method	OA (Tr = 50%)	OA (Tr = 80%)
AlexNet	Fine-tuning	–	96.67 ± 0.26%
VGG16	Fine-tuning	–	98.03 ± 0.26%
VGG16	VGG-M	–	97.42 ± 1.79%
AlexNet	MSCP	–	97.29 ± 0.63%
VGG16	MSCP	–	98.36 ± 0.58%
AlexNet	MCNN	–	96.66 ± 0.90%
AlexNet	DCNN	–	96.67 ± 0.10%
VGG16	DCNN	–	98.93 ± 0.10%
AlexNet	SCCOV	–	98.04 ± 0.23%
VGG16	SCCOV	–	99.05 ± 0.25%
VGG16	GBNet	97.05 ± 0.19%	98.57 ± 0.48%
VGG16	ARCNet	96.81 ± 0.14%	**99.12 ± 0.40%**
AlexNet	ADRL-Net	93.63 ± 0.13%	97.64 ± 0.34%
VGG16	ADRL-Net	**97.31 ± 0.13%**	98.14 ± 0.33%
ResNet50	ADRL-Net	**98.72 ± 0.12%**	**99.08 ± 0.31%**

**Table 3:** OA comparison of different methods on NWPU dataset. The best two results are highlighted in bold font.

Backbone	Method	OA (Tr = 10%)	OA (Tr = 20%)
AlexNet	Fine-tuning	80.66 ± 0.29%	84.74 ± 0.31%
VGG16	Fine-tuning	87.76 ± 0.10%	91.67 ± 0.12%
AlexNet	BoVW	55.22 ± 0.39%	59.22 ± 0.18%
VGG16	BoVW	82.65 ± 0.31%	84.32 ± 0.17%
AlexNet	MSCP	81.70 ± 0.23%	85.58 ± 0.16%
VGG16	MSCP	85.33 ± 0.21%	88.93 ± 0.14%
AlexNet	DCNN	85.56 ± 0.20%	87.24 ± 0.12%
VGG16	DCNN	89.22 ± 0.50%	91.89 ± 0.22%
AlexNet	SCCOV	84.33 ± 0.26%	87.30 ± 0.23%
VGG16	SCCOV	89.30 ± 0.35%	92.10 ± 0.25%
AlexNet	ADRL-Net	86.21 ± 0.25%	89.61 ± 0.22%
VGG16	ADRL-Net	**90.67 ± 0.24%**	**93.23 ± 0.23%**
ResNet50	ADRL-Net	**91.34 ± 0.24%**	**94.48 ± 0.21%**

**Table 4:** OA comparison of different methods on WHU-RS19 dataset. The best two results are highlighted in bold font.

Backbone	Method	OA (Tr = 40%)	OA (Tr = 60%)
VGG16	Fine-tuning	96.74 ± 0.57%	96.88 ± 0.61%
VGG16	DFF	–	98.65 ± 0.43%
VGG16	ARCNet	97.50 ± 0.49%	**99.75 ± 0.25%**
VGG16	GBNet	97.32 ± 0.32%	**99.25 ± 0.50%**
AlexNet	ADRL-Net	95.34 ± 0.37%	94.83 ± 0.26%
VGG16	ADRL-Net	**98.16 ± 0.35%**	99.05 ± 0.25%
ResNet50	ADRL-Net	**98.70 ± 0.36%**	**99.86 ± 0.26%**

## Data Availability

The AID dataset in this paper comes from Xia, G.-S.; Hu, J.; Hu, F.; Shi, B.; Bai, X.; Zhong, Y.; Zhang, L.; and Lu, X. 2017. A benchmark data set for performance evaluation of aerial scene classification. IEEE Transactions on Geoscience and Remote Sensing 55(7): 3965–3981. https://doi.org/10.1109/TGRS.2017.2685945. The UC Merced dataset in this paper comes from http://vision.ucmerced.edu/datasets/, accessed on 27 November 2022. The NWPU dataset in this paper comes from Cheng, G.; Li, Z.; Yao, X.; Guo, L.; and Wei, Z. 2017. Remote sensing image scene classification using bag of convolutional features. IEEE Geoscience and Remote Sensing Letters 14(10): 1735–1739. https://doi.org/10.1109/JPROC.2017.2675998. The WHU-RS19 dataset in this paper comes from Dai, D.; and Yang, W. 2010. Satellite image classification via two-layer sparse coding with biased image representation. IEEE Geoscience and Remote Sensing Letters 8(1):173–176. https://doi.org/10.1109/LGRS.2010.2055033.
